# Protein Kinases in Leukemias

**DOI:** 10.3390/cancers13112747

**Published:** 2021-06-01

**Authors:** Paulo De Sepulveda, Jean-Max Pasquet

**Affiliations:** 1 Cancer Research Center of Marseille, CRCM, INSERM U1068, UMR7258 CNRS, Aix-Marseille Université, Institut Paoli-Calmettes, 13009 Marseille, France; 2 INSERM U1035 BMGIC, Université de Bordeaux, 33076 Bordeaux, France

Protein kinases (PK) make up around 2% of the human genome and their expression profile varies depending on the organ and tissue. They play crucial roles in intracellular signaling, thus controlling cell behavior such as proliferation, differentiation, mobility or metabolism. Therefore, the enzymatic activities of PK are severely regulated as they control homeostasis and cell fate. Many PK are therapeutic targets because they participate in signaling pathways which, once deregulated, lead to pathologies, and in particular cancer. As such, several PKs have been identified as deregulated in various hemopathies including leukemias. Indeed, deregulation of expression and/or activity of kinases through mutations or other mechanisms leads to a wide range of diseases and cancers. Blocking the enzymatic activity therefore became a quest for pharmaceutical companies. Around 50 kinase inhibitors are currently FDA-approved while at least 150 are being investigated in clinical trials.

Iconic PK involved in hematopoiesis and also in leukemia include the receptors KIT and FLT3, and the non-receptor tyrosine kinases ABL and JAK2. These four PK are major oncoproteins and therapeutic targets frequently mutated in specific malignant hemopathies. In addition, many other PK, whether mutated or not, fuel oncogenic signaling pathways that participate to the maintenance and regeneration of leukemic state.

This Special Issue of *Cancers*, “PKs in leukemia”, had as objectives to present various clinical and biological aspects, but also to remind that PK pharmacological targeting is both of great interest and sometimes complicated or difficult to implement when harmful side effects occur. In this Special Issue, the benefit of inhibiting several kinases is reported in particular in chronic myelogenous leukemia (CML) to reduce the pool of residual leukemic stem cells [1]. However, therapeutic targeting by inhibition of PK may also be accompanied by adverse effects, as reported in CML for cardiac cytotoxicity [2]. In contrast, some PK inhibitors, such for Btk, have also been proposed as antithrombotic drugs [3]. In parallel, the use of a PK inhibitor in acute myeloblastic leukemia (AML) FLT3-ITD or acute lymphoblastic leukemia (ALL) Ph^+^ demonstrates a broad benefit in terms of patient management and toxicity but also reveals new resistance as well as unchanged long-term survival [4,5,6]. 

In addition, targeting PK in various hematologic malignancies such as myeloma may be necessary when resistance to corticoid treatment develops. However, it should be kept in mind that the more specific the targeting, the more resistance mechanisms may emerge; therefore, new pharmacological approaches are required, such as the use of withaferin A, a promiscuous kinase inhibitor in glucocorticoid resistance in myeloma [7]. 

The development of PK inhibitors in the treatment of hematologic malignancies such as leukemia emerged with imatinib in CML 20 years ago. Over the last two decades, many improvements have been made to introduce new pharmacological inhibitors in CML, CLL and AML [5,8,9,10]. This is illustrated by the seven reviews of this Special Issue, with a panel of the different mechanisms and signaling pathways involved in the response or resistance to these treatments. The articles exemplify the interest of targeting a protein kinase when it plays an oncogenic role but also its upstream or downstream signaling to increase the chances of success in treatments as reported in CML and CLL [10]. We will also see that many other PK can be targeted jointly or sequentially to avoid the emergence of resistance or increase the therapeutic potential of the treatments. We can then use combinations or multi-target inhibitors to increase [1,4,8]. 

Indeed, combination therapy has several advantages. First, targeting two independent molecular pathways leaves less opportunity for escape mechanisms, illustrated by the crosstalk between kinases and phosphatases in CML [11,12]. Second, if the molecules used in combination show synergy, they can then be used with efficacy at lower doses, which means lower secondary effects for patients. Finally, considering the heterogeneity of leukemia samples, combination therapy is more likely to benefit to large number of patients than single therapy with PK inhibitors [1,8,12].

These reviews confirm the enormous beneficial advances of PK-targeted treatments. We can also transpose the use of these molecules in other pathologies such as thrombotic pathologies. In conclusion, this Special Issue devoted to PK in leukemia illustrates that the development of PK therapeutic targeting is still an ongoing process, and that the notion of combination therapy and administration window may soon become the standard in clinical practices.
